# Bone Regeneration from PLGA Micro-Nanoparticles

**DOI:** 10.1155/2015/415289

**Published:** 2015-10-05

**Authors:** Inmaculada Ortega-Oller, Miguel Padial-Molina, Pablo Galindo-Moreno, Francisco O'Valle, Ana Belén Jódar-Reyes, Jose Manuel Peula-García

**Affiliations:** ^1^Department of Oral Surgery and Implant Dentistry, University of Granada, 18011 Granada, Spain; ^2^Department of Pathology, School of Medicine and IBIMER, University of Granada, 18012 Granada, Spain; ^3^Biocolloid and Fluid Physics Group, Department of Applied Physics, University of Granada, 18071 Granada, Spain; ^4^Department of Applied Physics II, University of Málaga, 29071 Málaga, Spain

## Abstract

Poly-lactic-co-glycolic acid (PLGA) is one of the most widely used synthetic polymers for development of delivery systems for drugs and therapeutic biomolecules and as component of tissue engineering applications. Its properties and versatility allow it to be a reference polymer in manufacturing of nano- and microparticles to encapsulate and deliver a wide variety of hydrophobic and hydrophilic molecules. It additionally facilitates and extends its use to encapsulate biomolecules such as proteins or nucleic acids that can be released in a controlled way. This review focuses on the use of nano/microparticles of PLGA as a delivery system of one of the most commonly used growth factors in bone tissue engineering, the bone morphogenetic protein 2 (BMP2). Thus, all the needed requirements to reach a controlled delivery of BMP2 using PLGA particles as a main component have been examined. The problems and solutions for the adequate development of this system with a great potential in cell differentiation and proliferation processes under a bone regenerative point of view are discussed.

## 1. Introduction

Bone regeneration is one of the main challenges facing us in the daily clinic. Immediately after a tooth extraction, normal biological processes remodel the alveolar bone limiting in some cases the possibility of future implant placement. Different strategies for the preservation of that bone have been explored in recent years. Other conditions, such as trauma, tumor resective surgery, or congenital deformities, require even higher technical and biological requirements to generate the necessary bony structure for the occlusal rehabilitation of the patient. To overcome these anatomical limitations in terms of bone volume, different approaches have been proposed to either improve the implant osteointegration or to augment the bone anatomy where it will be placed [1, 2]. Autogenous bone graft is still considered the “gold standard” due to its osteogenic, osteoconductive, and osteoconductive properties [3, 4]. However, it also presents several limitations including the need for a second surgery, limited availability, and morbidity in the donor area [5]. Therefore, other biomaterials such as allogeneic grafts, with osteoconductivity and osteoinductive capacities [6, 7], and xenogeneic grafts [8, 9] and alloplastic biomaterials [10], with osseoconductive potential, were proposed. All these materials, although acceptable, are not suitable in many conditions and usually require additional consideration in the decision process [11]. Additionally, the bone quantity and quality that can be obtained with these materials are often limited.

The use of bioactive molecules, alone or in combination with the previously described materials, has, therefore, become a major area of interest thanks to their high potential. When using this kind of procedures, it is important to consider (1) the delivery method and (2) the molecule itself. Bioactive molecules can be transported into the defect area as a solution or a gel, embedded in sponges, adhered to solid scaffolds and, more recently, included in particles of different sizes. Using these methods, PDGF (platelet-derived growth factor), FGF (fibroblast growth factor), IGF (insulin growth factor), Runx2, Osterix (Osx), LIM domain mineralization protein (LMP), BMP (bone morphogenic protein) and, more recently, periostin have been proposed as potential candidates for regeneration procedures within the oral cavity, including bone and periodontal tissues [12, 13]. These molecules have been tested alone or in combination with stem cells [14] using several* in vitro* and* in vivo* strategies [15].

Consequently, within the context of this review, we intend to review the delivery methods of bioactive molecules with the purpose of bone regeneration, with a particular focus on polymeric nano/microparticles, especially those with PLGA as main component, to encapsulate the growth factor BMP-2. An overview of the biological functions of bone morphogenetic proteins and an analysis of the different parameters affecting the physicochemical properties of these systems are presented. Synthesis method, particle size and morphology, use of stabilizers and their incidence in the colloidal stability, protective function, and surface functionality will be discussed. In addition, we explore the different strategies that can be used to optimize the encapsulation efficiency and release kinetics, main parameters that determine the correct development of polymeric carriers used in tissue-engineered bone processes.

## 2. BMPs: Action and Regulation

For bone regeneration, in particular, bone morphogenetic growth factors (BMP) are probably the more tested group of molecules. Since 1965, when Urist [16] showed that the extracted bone BMPs could induce bone and cartilage formation when implanted in animal tissue, an increasing number of reports have tested its* in vivo* application and biological foundation when used in bone defects [17–19]. BMPs are members of the TGF-*β* superfamily of proteins [20]. The BMP family of proteins groups more than 20 homodimeric or heterodimeric morphogenetic proteins, which functions in many cell types and tissues, not all of them being osteogenic [21]. BMPs can be divided into 4 subfamilies based on their function and sequence, being BMP-2, BMP-4, and BMP-7 the ones with osteogenic potential [21]. The actions of BMPs include chondrogenesis, osteogenesis, angiogenesis, and extracellular matrix synthesis [22]. Within this family of proteins, BMP-2 has been the most studied. It has osteoinductive properties that promote the formation of new bone by initiating, stimulating, and amplifying the cascade of bone formation through chemotaxis and stimulation of proliferation and differentiation of the osteoblastic cell lineage [5, 17, 19, 20]. The absence of it, as studied in knockout models, leads to spontaneous fractures that do not heal with time [23]. In fact, other models have demonstrated that the absence of either BMP-4 [24] or BMP-7 [25] do not lead to bone formation and function impairment which demonstrate the compensatory effect produced by BMP-2 alone [26].

Many cell types in bone tissue produce BMPs, including osteoprogenitor cells, osteoblasts, chrondrocytes, platelets, and endothelial cells. This secreted BMP is then stored in the extracellular matrix where it mostly interacts with collagen type IV [27]. During the repair and remodeling processes, osteoclast resorptive activity induces the release of BMPs to the medium so that they are suspended and can interact with nearby cells to initiate the subsequent osteogenic process [28].

**Figure 1:**
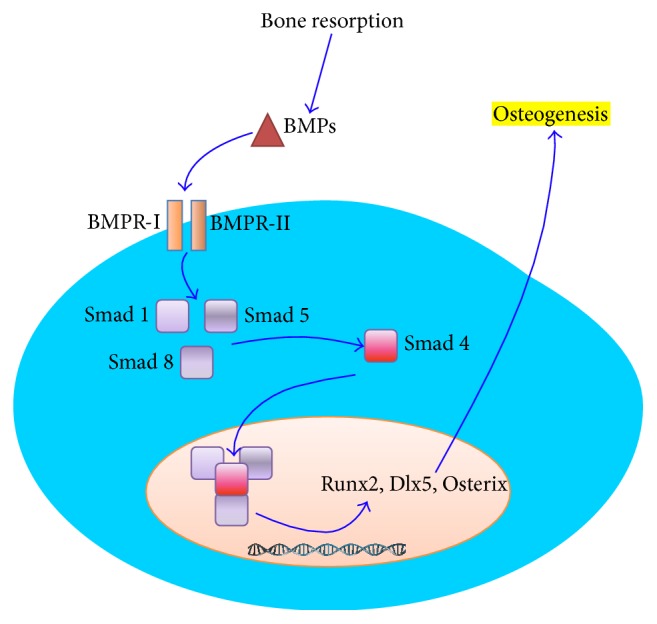
Schematic representation of the main BMP molecular pathway to osteogenesis. BMPs interact with cell surface receptors I and II to activate Smads 1, 5, and 8. These activated Smads activate Smad 4. All together as a protein complex activate Runx2, Dlx5, and Osterix.

A BMP in the extracellular matrix binds to cell surface receptors BMPR-I and BMPR-II and activates the Smad cytoplasmic proteins or the MAPK pathway [29]. When BMPR-I is activated, BMPR-II is recruited and activated as well [30]. The activation of the complexes BMPR-I and BMPR-II leads to the activation of several Smads (1, 5, and 8) that also activate Smad 4 and they all form protein complexes that are transported into the nucleus where Runx2, Dlx5, and Osterix genes (important in osteogenesis) are activated [26, 27] (Figure 1). Similarly, when the MAPK pathway is activated, it leads to induction of Runx2 transcription and, therefore, to bone differentiation [31]. A number of extracellular and intracellular antagonists have also been described, including noggin, chordin, and gremlin or Smads 6, 7, and 8b, respectively [32].

### 2.1. Clinical Use of BMP-2

Today, the BMP-2 is commercially available under different brand names and concentrations. It usually consists of a collagen absorbable sponge embedded with recombinant human BMP-2. In 2002, it was approved by the FDA as an alternative of autogenous bone grafting in anterior lumbar interbody fusion [33]. Later, in 2007, the FDA approved the use of rhBMP-2 as an alternative for autogenous bone grafting in the increase of the alveolar crest defects associated with the tooth extraction maxillary sinus pneumatization [33].

Beside the applications in spine clinical studies, where very high concentrations are used (AMPLIFY, rhBMP-2, 40 mg), clinical studies have supported its use in the oral cavity. BMPs have been used in periodontal regeneration, bone healing, implant osteointegration, oral surgery with orthodontic purposes, bone pathology sequel repair, distraction osteogenesis, and endodontic reparative surgery [28, 34]. However, it has shown more promising results in cases where only bone tissue is to be regenerated, including preimplant site development, sinus lift, vertical and horizontal ridge augmentation, and dental implant wound healing [35]. In this sense, it has been shown that the use of rhBMP-2 induced the formation of bone suitable for placement of dental implants and their osteointegration [36]. Furthermore, it appears that the newly formed bone has similar properties to the native bone and is, therefore, capable of supporting denture occlusal forces [37]. In the particular case of sinus lifting, where bone deficiency is greater and, therefore, supportive therapies can be more helpful, a recent meta-analysis found a total of 3 human studies and 4 animal trials (Table 1) [38]. In summary, the included studies concluded that rhBMP-2 induces new bone formation with comparable bone quality and quantity of newly formed bone to that induced by autogenous bone graft. In some cases, even higher bone quality and quantity have been reported [39].

Conversely, recent studies report severe complications after its use [61]. Even more, high doses have also associated with carcinogenic effects, which led the authors to emphasize the need for better guidelines in BMP clinical use [62]. Not so drastic, recent studies are highlighting the negative side effects and risks of its application, making high emphasis on potential bias of nonreproducible industry sponsored research, especially when used in spinal fusion [44, 63, 64]. The use of rhBMP-2 has been shown to increase the risks for wound complications and dysphagia with high effectiveness and harms misrepresentation through selective reporting, duplicate publication, and underreporting [44]. Specifically in oral bone regenerative applications, a report in sinus lift concluded that the use of BMP-2 promotes negative effects on bone formation when combined with anorganic bovine bone matrix versus anorganic bovine bone alone [41], in contrast with previous reports and reviews [38]. Taking together this information, it can be concluded that it is of extreme importance to be careful with the clinical use of new products, avoiding off-label applications. It is also important to highlight the need for more and better clinical research.

To overcome these limitations, new strategies, such as the use of* ex vivo* BMP-2-engineered autologous MSCs [65], encapsulation of the protein in different biomaterials, or delivery by gene therapy, are being explored in recent years.

The development of these technologies is based on some biological facts.* In vitro* effects of BMPs are observed at very low dosages (5–20 ng/mL), although current commercially available rhBMPs are used in large dosages (up to 40 mg of some products) [28]. This is probably due to an intense proteolytic consumption during the early postsurgical phases. It is important to know the proper sequence of biological events that lead to normal tissue healing. Then, this knowledge can be used to intervene at the specific time frame where our therapy is intended to act [15]. Effective bone formation, as described above, is a sequential process. Therefore, the inductive agent should be delivered at a maintained concentration during a timeframe. In this sense, as in many other processes in medicine, it has been recently demonstrated that long-term release of BMP-2 is more effective than short-term over a range of doses [51]. It is also important to note that the role of other molecular pathways and crosstalk between the different components playing in bone regeneration is not perfectly understood yet, and, therefore, more research has to be conducted.

What is known so far, in summary, is that BMPs, specifically BMP-2, is of utility for promoting bone regeneration [28]. However, the currently FDA-approved BMP-2 delivery system (INFUSE, Medtronic Sofamor Danek, Inc.) presents important limitations [66]. Firstly, protein is quickly inactivated. Therefore, its biological action disappears, maybe even before the blood clot that forms after the surgery is being organized. Second, the recombinant protein is delivered in an absorbable collagen sponge. Thus, the distribution of the BMP in a liquid suspension embedded into a collagen sponge makes it impossible to be certain that the protein is reaching the ideal target. Therefore, where, when, and for how long a dose of BMP-2 is reached (determined by the delivery method) are important factors. Because of that, new forms of BMP-2 delivery are being developed. These new technologies have to guarantee a higher half-life of the protein and a stepped release, to increase the effects on the desired cell targets. The biotechnology opens the door to be able to provide a solution to these limitations.

Biodegradable nanoparticles (nanospheres and nanocapsules) have developed as a promising important tool for the delivery of macromolecules via parenteral, mucous, and topical applications [67–70]. Well-established biodegradable polymers such as poly(acid D, L-lactic) or poly(D, L-lactic-co-glycolic) have been widely used in the preparation of nanoparticles in recent decades because of its biocompatibility and full biodegradability [71]. However, it is known that certain macromolecules, such as proteins or peptides, may lose activity during their encapsulation, storage, delivery, and release [72]. To overcome this problem, the addition of stabilizers such as oxide polyethylene (PEO) or the coencapsulation with other macromolecules and its derivatives seem to be a promising strategy.

## 3. Polymeric Colloidal Particles to Encapsulate Hydrophilic Molecules

Generally, polymeric colloidal particles are hard systems with a homogeneous spherical shape composed by natural or synthetic polymers. In order to encapsulate hydrophilic molecules as proteins or nucleic acids, it is necessary to optimize the polymeric composition and the synthesis method. In this process, a high encapsulation efficiency, maintenance of the biological activity of the encapsulated biomolecule, and obtaining of an adequate release pattern have to be achieved [73–75]. Several delivery systems of BMP2 (and other growth factors, GFs) using polymeric particles have been described in the literature. Most of them are microparticulated systems using the biocompatible and biodegradable PLGA copolymer as main component [76, 77]. Taking into account the incorporation of BMP2 to the carrier system, encapsulation is preferred to absorption because the growth factors are more protected against environmental factors in the medium and may have better control over the delivery and release to achieve the desired concentrations in specific site and time [78].

Normally, if the GFs are related with bone regeneration processes, nano-microparticles are trapped in a second system as hydrogels or tissue engineering scaffolds, which also play an important role in the release profile of GFs from these particles [78]. The nano-microparticles have allowed the development of multiscale scaffold, thereby facilitating control of the internal architecture and adequate patterns of mechanical gradients of cells and signaling factors [79].

All steps, from the synthesis method and its characteristics, the encapsulation process, or the final surface modification for a targeted delivery, determine the characteristics of these systems and their main goal: the controlled release of bioactive GFs.

### 3.1. Synthesis Methods

**Figure 2:**
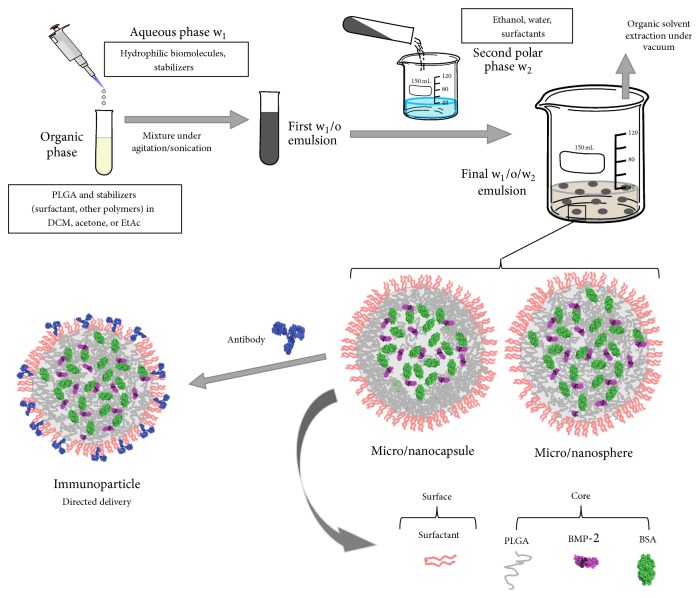
Double emulsion procedure (water/oil/water emulsion, W_1_/O/W_2_) to obtain PLGA micro/nanoparticles. Depending on the synthesis conditions (stabilizers, solvents and mixing procedure) it is possible to obtain micro-nanospheres with a uniform matrix or micro-nanocapsules with a core-shell structure. Immunoparticles used for directed delivery can be obtained by attaching specific antibody molecules on the particle surface.

It is possible to found several procedures to encapsulate hydrophilic molecules as proteins or nucleic acids in polymeric nano/microparticles. Phase separation [80] or spray drying [81] techniques have been reported to encapsulate hydrophilic molecules. However, in the case of proteins, the most normally used procedure to encapsulate them into PLGA micro- and nanoparticles is the double-emulsion (water/oil/water, W/O/W) solvent evaporation technique [75, 82]. A schematic description of this technique is presented in Figure 2. In a general way, PLGA is dissolved in an organic solvent and emulsified, using mechanical agitation or sonication, with water containing an appropriate amount of protein. Thus, a primary water/oil (W/O) emulsion is obtained. In the second phase, this emulsion is poured into a large polar phase leading to an immediate precipitation of the particles as a consequence of the polymer shrinkage around droplets of the primary emulsion. This phase may be composed of a water solution of a stabilizer (surfactant) or ethanol-water mixtures [83, 84]. After stirring, the organic solvent is rapidly extracted by evaporation under vacuum. A wide list of different modifications have been tested in this procedure in order to obtain a micro/nanocarrier system with adequate colloidal stability, high encapsulation efficiency, adequate bioactivity, and, finally, a long-time release profile with low “initial burst.” The goal is to avoid a high amount of protein (>60%) being released very quickly (24 hours), which is one of the biggest problems of a controlled release system [76].

### 3.2. Organic Solvent

Hans and Lowman show different examples of organic solvents used in multiple emulsion processes. Normally, dichloromethane (DMC), ethyl acetate, acetone and their mixtures can be used [82]. In the first step, a good organic solvent with low water solubility to facilitate the emulsification process and low boiling point for an easy evaporation would be the election. However, the structure of the encapsulated protein molecules can be affected and denaturation processes and loss of biological activity appear when they interact with a typical organic solvent as DMC [73]. Ethyl acetate, on the other hand, exerts less denaturating effects with a lower incidence on the bioactivity of the encapsulated proteins [85].

Other important factors related with the organic solvent are their physical properties that affect how the polymer tails self-organize in the shell of the emulsion droplets and modify the nanoparticle morphology and the encapsulation efficiency [86]. In this way, a higher water solubility of the organic solvent, that is, ethyl acetate, favors a rapid solvent removal. Additionally, the solvent removal rate can be controlled by adjusting the volume of the polar phase as well as the shear stress during the second emulsification step. An increase of these two parameters increases the diffusion rate of ethyl acetate from primary microparticles to outer aqueous phase, resulting in their rapid solidification [87]. It also enhances the encapsulation efficiency and minimizes the contact-time between protein molecules and organic solvent [88], obtaining at the same time a lower burst effect and a slower drug release from the microparticles [87].

### 3.3. Particle Size and Morphology

Particle size is an important parameter and one of the main goals of the delivery polymeric system. Microspheres, from a few micrometers up to 100 *μ*m, are suitable for oral delivery, mucosal adhesion, or inside scaffold use, that is, for bone regeneration. Nanoscale dimension of the carrier offers enhanced versatility when compared with particles of larger size. This is due to the fact that they have higher colloidal stability, improved dispersibility and bioavailability, more reactive surface and also, can deliver proteins or drugs inside and outside of the corresponding cells [89]. BMP2 promotes bone formation and induces the expression of other BMPs and initiates the signaling pathway from the cell surface by binding to two different surface receptors [22]. Therefore, the BMP2 carrier particles must release it into the extracellular medium. Since cellular intake of PLGA nanoparticles is very fast, the intaking process can be limited by an increase in size from nano- to microparticles [90]. However, the interaction between particles and cells is strongly influenced by particle size. If cell internalization is desired, the particle must be comprised in the submicron scale at an interval between 2 and 500 nm [91]. Moreover, this size is needed for a rapid distribution after parenteral administration in order to reach different tissues through different biological barriers. In addition, the intake by macrophages is minimized with a diameter of nanoparticles under 200 nm and even smaller [82, 92]. As discussed by Yang et al. [93], slight modifications of the synthesis procedure can suppose drastic effects on the size or particle morphology and, therefore, in the protein encapsulation efficiency and kinetic release.

In double emulsion processes, the first emulsification step largely determines the particle size while the second emulsification step, characterized by the solvent elimination and polymer precipitation, mainly affects the particle morphology [86]. However, the use of surfactant solutions as the polar medium of the second emulsification process and the volume ratio between organic and polar phases in this step has shown an important influence in the final size [94]. Therefore, the correct election of the organic solvent, the polymer concentration, the addition of surfactant, and the emulsification energy allow controlling the size of the system.

**Figure 3:**
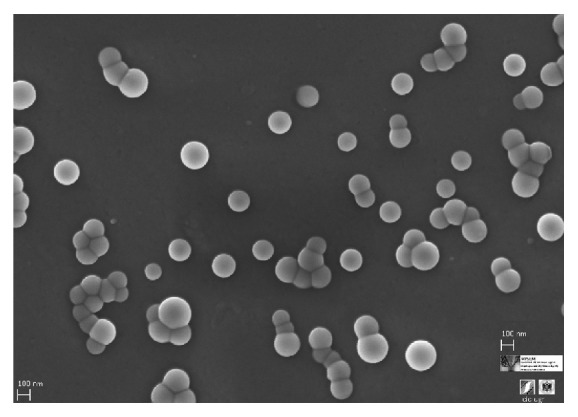
Scanning electron microscopy (SEM) photography of PLGA nanoparticles obtained by a double emulsion emulsification procedure. This system with spherical shape, low polydispersity, and nanoscopic scale shows the intended properties for an adequate physiological distribution and cell internalization.

**Figure 4:**
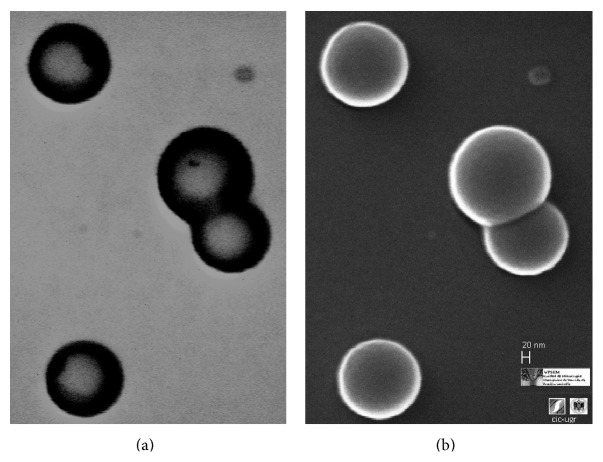
PLGA/poloxamers188 blend nanoparticles. (a) Scanning transmission electron microscopy (STEM) photography; (b) scanning electron microscopy (SEM) photography. STEM technique allows the analysis of the nanoparticle structure with an internal region with a low polymer density, which is representative of nanocapsules with core-shell structure.

The incorporation of poloxamers (F68) in the organic solvent of the primary emulsification helps to increase the colloidal stability of the first dispersion by being placed at the water/oil interface. This reduces the particle size in comparison with pure PLGA nanoparticles in which the only stability source comes from electric charge of the carboxyl groups of the PLGA [95]. It is normal to obtain spherical micro/nanospheres with a polymeric porous core. A typical SEM micrograph of PLGA nanoparticles obtained by W/O/W emulsion using a mixture of organic solvents (DCM/acetone) and ethanol/water as second polar medium is shown in Figure 3, in which the spherical shape and uniform size distribution are the main characteristics. The outer polymeric shell in the second emulsification step pushed the water droplets to the inner core according to their solidification process [96]. This process allows producing particles like capsules with a core-shell structure in which the inner core has a low polymer density.  Figure 4 shows a typical core-shell structure in which the polymer precipitates and shrinks around the water droplets during the solvent change of the second phase and the subsequent organic solvent evaporation process [97]. In this case, the process of solidification of the polymer is influenced and determined by the miscibility of the organic solvent with the second polar phase and the removal rate.

The polymeric shell often presents channels or pores as a consequence of the inner water extrusion due to osmotic forces. This can reduce the encapsulation efficiency and favors a fast initial leakage with the unwanted “burst release” [93]. This modification of internal structure of the particles is usually indicated assigning the term “nanosphere” to the system with a core consisting of a homogeneous polymer matrix. The bioactive agent is dispersed within them, while the core-shell structure would be similar to a “nanocapsule” where the biomolecule is preferably in the aqueous cavity surrounded by the polymeric shell [78] (see Figure 2).

### 3.4. Stabilizer Agents

#### 3.4.1. Colloidal Stability

The double emulsion method normally requires the presence of stabilizers in order to confer colloidal stability during the first emulsification step, to prevent the coalescence of the emulsion droplets, and, later, to maintain the stability of the final nano/microparticles [98]. Polyvinyl alcohol (PVA) and PEO derivate as poloxamers (also named pluronics) have been used in most cases [83, 94]. Others include natural surfactants, such as phospholipids [99, 100]. In some cases, it is possible to avoid surfactants if the particles have an electrostatic stability contribution, that is, from the uncapped end carboxyl groups of the PLGA molecules [101].

As it has been previously commented, PVA and poloxamers have shown their efficiency in synthetizing both nano- and microparticles, affecting not only the stability of the systems but also their size and morphology. Thus, a size reduction effect has been found using PVA in the external water phase, affecting at the same time the surface porosity, mainly in microsized particles [94]. A comparative study between this and phospholipids (di-palmitoyl phosphatydilcholine, DPPC) as stabilizers showed that DPPC could be a better emulsifier than PVA to produce nano- and microparticles. With this method, a much lower amount of stabilizer was needed to obtain a similar size. In the same study, a higher porosity on the particle surface for the PVA emulsified nanospheres was shown [99].

**Figure 5:**
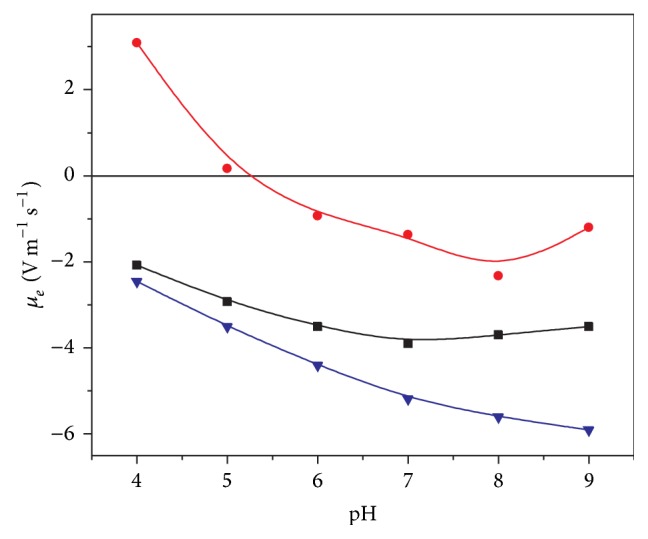
Electrophoretic mobility versus pH for PLGA nanoparticles with different characteristics. (▼) PLGA, (■) PLGA/poloxamer188 blend, and (∙) PLGA covered by Immuno-*γ*-globulin. The different surface composition affects the electrokinetic behaviour of bare nanoparticles. Surface charge values were screened by the presence of nonionic surfactant as poloxamers, or, in a higher extension, by the presence of antibody molecules attached on the surface.

On the other hand, the combination of PLGA with poloxamers has shown positive effects for the nano- and microsystems in terms of stability [102]. The use of these surfactants in the first or second steps of the W/O/W emulsion procedure leads to different situations. Thus, if poloxamers are blended with PLGA in the organic phase of the primary emulsification, an alteration of the surface roughness is obtained. However, if these are added in the inner water phase, an increase of porosity is found [83]. In addition, their inclusion in the polar phase of the second emulsification step also generates hydrophilic roughness surfaces. A quantification of this is shown in Figure 5, in which the electrophoretic mobility of both PLGA pure and PLGA/pluronic F68 nanoparticles is measured as a function of the pH of the medium. The observed dependence with this parameter is a consequence of the weak acid character of the PLGA carboxyl groups. When poloxamer molecules are present at the interface, a systematic reduction of mobility was found as a consequence of the increase in the surface roughness. The hydrophilic surfactant chains spread out towards the solvent originating a displacement of the shear plane and the consequent mobility reduction [95, 101].

The final PLGA particle size is primarily controlled by electrostatic forces and is not significantly affected by the presence or nature of poloxamer stabilizers [101]. The recognition of the nanocarriers by the mononuclear phagocytic system (MPS) can be significantly altered if the surface of colloidal particles is modified by using PEO block copolymer of the poloxamer molecules. The steric barrier given by these surfactant molecules prevents or minimizes the adsorption of plasma protein and decreases the recognition by macrophages [103]. The size of microspheres is also unaffected by the coencapsulation of poloxamers. The system containing poloxamer-PLGA blends drive to an inner structure displaying small holes and cavities in relation with microspheres of pure PLGA with a compact matrix-type structure [83].

Microparticles formulated by poloxamer in the second polar medium have completely different surface than the PVA ones, almost without pores [94]. A comparison between different poloxamers shows that the hydrophilic-lipophylic balance (HBL) of the surfactant plays a crucial role determining the surfactant-polymer interactions and controlling the porosity and roughness of the nano-microparticles [83, 104].

In a similar manner to surfactants, polymer characteristics, like the hydrophobicity grade, the molecular weight or the hydrolysis degradation rate, can strongly influence the particle morphology. Therefore, the polymer composition of the particles greatly affects its structure and properties. This is why it is usual to use other polymers in order to modify the behavior and application of the particles. In this way, polyethylene glycol (PEG) of different chain length is frequently used to modify the surface characteristics. With PEG, particles are more hydrophilic and with rougher surfaces which affects the MPS action by increasing the circulating-time and half-life* in vivo*, like the presence of PEO chains [105]. Additionally, PEG chains also provide colloidal stability via steric stabilization. Pegylated-PLGA nano- or microparticles can be normally obtained by using in the synthesis method PLGA/PEG di- and triblock copolymers [58, 59, 75]. Natural polymers as chitosan, besides modifying the hydrophobicity-hydrophilicity ratio of the surface, also confer them a mucoadhesive character [106].

#### 3.4.2. Encapsulation Efficiency and Bioactivity

Furthermore, the use of stabilizers (surfactants or polymers) also influences the encapsulation efficiency and the protein stability. In fact, for the W/O/W solvent evaporation process, the chlorinated organic solvent used for the first emulsification could degrade protein molecules encapsulated in this step if they come into contact with the organic/water interface, causing their aggregation or denaturation [107]. The polymer-protein interaction, the shear stress for the emulsification process, and the pH reduction derived from PLGA polymer degradation can also produce the same situation with the subsequent loss of biological activity of the encapsulated biomolecules. Different strategies to prevent it have been used. For example, an increase of the viscosity around protein molecules can help to isolate them from their microenvironment [108]. In this way, viscous products, such as starch, have been used to prevent protein instability [109]. These authors coencapsulate BMP2 with albumin inside starch microparticles using other biodegradable polymer, poly-*ε*-caprolactone, instead of PLGA. The BMP2 retained its bioactivity. Despite a low encapsulation rate, beside an initial burst followed by an uncompleted release, the amount of BMP2 needed at the beginning was lower [109]. The combination of PEO surfactants with PLGA (blended in the organic phase) can also preserve the bioactivity of microencapsulated proteins [110] or nucleic acids [84].

However, in most cases, the coencapsulation of GFs with other biomolecules was the preferred strategy. Thereby, serum albumins (SA) have shown the capacity to limit the aggregation-destabilization of several proteins incited by the water/organic solvent interface of the primary emulsification process [111, 112]. White et al. encapsulated lysozyme inside PLGA-PEG microparticles. In addition to the protective function, they also observed an important increase of the entrapment efficiency when human SA was coencapsulated with lysozyme and BMP2 [59]. d'Angelo et al. used heparin as stabilizer because it forms a specific complex with several GFs, stabilizes their tridimensional structure, and promotes their bioactivity. An encapsulation efficiency of 35% was increased to 87% using bovine SA as a second stabilizer to encapsulate two natural proangiogenic growth factors inside PLGA-poloxamer blended nanoparticles. The* in vitro* cellular assays showed the preservation of the biological activity of GFs up to one month [56].

The use of more hydrophilic surfactants (poloxamers) or polymers (PEG) in the inner water phase or blended with PLGA in the organic phase of the primary emulsion reduces the interaction of encapsulated proteins with the hydrophobic PLGA matrix. This prevents disrupting the structure of the protein molecules and helps, at the same time, to neutralize the acidity generated by the hydrolytic degradation of the PLGA [113]. In some cases, the combination of several stabilizers, such as poloxamers, trehalose, and sodium bicarbonate, has been shown to preserve the integrity of encapsulated proteins but it also reduces the encapsulation efficiency [114].

As a general rule, encapsulation efficiency increases with the size of the particles [82]. Additionally, the adequate stabilization of the primary emulsion by amphiphilic polymers and a rapid solidification (precipitation) of polymer in the second step are favorable parameters for enhancing protein entrapment efficiency in the W/O/W emulsion technique [87].

The tendency of BMP2 to interact with hydrophobic surfaces may decrease the loss of encapsulated protein during the extraction of the solvent phase. This favors a higher entrapment but it lowers the later extraction [58]. An optimal protein encapsulation is obtained when pH of the internal and external water phases is near the isoelectric point of the protein [92]. Blanco and Alonso [83] observed a reduction in the protein encapsulation efficiency when poloxamer was coencapsulated in the primary emulsion. This highlights the main role played by the protein-polymer interaction in the encapsulation efficiency and the later release process. However, too much emulsifier may also result in a reduction of the encapsulation efficiency [99]. Therefore, an equilibrium between the emulsification powder of the surfactant and their concentration is needed.

### 3.5. Release Profile

The release profile represents one of the most important characteristics of a nano/micro particulate carrier system since their development has a main final objective: the adequate release of the encapsulated bioactive molecules to reach the desired clinical action.

**Figure 6:**
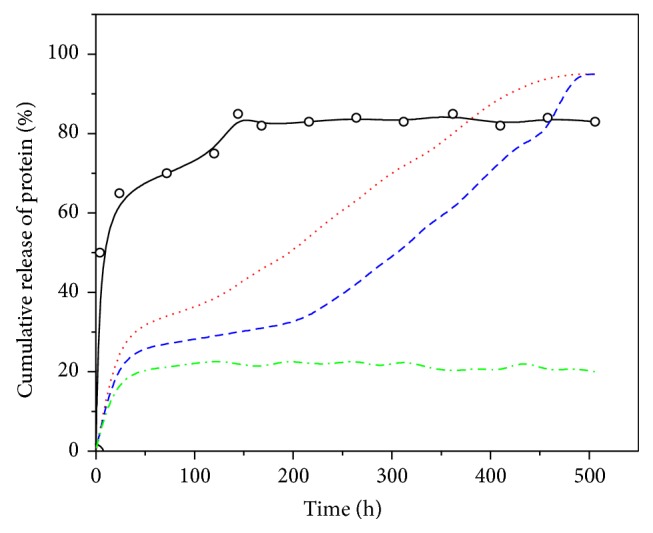
Release profiles. (○) BSA release from PLGA nanoparticles with high initial burst release; (red dots line) biphasic model combining a moderate initial burst and a subsequent sustained release; (blue dash line) triphasic model with a lag of release between both initial and sustained release phases; (dash-dot green line) incomplete release.

The release pattern of protein encapsulated in PLGA micro/nanoparticles can present different behavior. It is possible to find a continuous release when the diffusion of the biomolecule is faster than the particle erosion. This process involves a continuous diffusion of the protein from the polymer matrix before the PLGA particle is degraded in lactic and glycolic acid monomers by hydrolysis [74]. A biphasic release characterized by an initial burst at or near the particle surface followed by a second phase in which protein is progressively released by diffusion has also been described. The second phase can be enhanced by bulk erosion of PLGA shell and matrix which results in an important increase of pores and channels [75]. A third triphasic release profile has been found when a lag release period occurs after initial burst and until polymer degradation starts [115]. Finally, it is possible to obtain an incomplete protein release as a consequence of additional factors related with the protein-polymer interaction or protein instability.  Figure 6 illustrates the different release profiles previously described. The optimal carrier system should be capable of releasing a controlled concentration gradient of growth factors in the appropriate time, preventing or at least reducing or controlling the initial burst effect [116]. A controlled initial burst followed by a sustained release significantly improves the* in vivo* bone regeneration [117–119].

Giteau et al. [108] present an interesting revision on “How to achieve a sustained and complete release from PLGA microparticles.” They begin by analyzing the influence of the release medium and sampling method on the release profile and highlight the significance of the centrifugation cleaning process or the release medium volume. Adjusting to adequate values the centrifugation speed or the buffer volume, it is possible to separate micro/nanoparticles from protein-containing release medium in a very easy way. This allows for stable and reproducible release patterns. On the other hand, to ensure a better protein release profile, modification of the microparticle formulation and microencapsulation process in order to preserve protein aggregation has to be performed. Protein stability has to be maintained by preventing the formation of harmful medium. For example, the synthesis formulation can be modified to use more hydrophilic polymers, since they have been shown to reduce the initial burst and to deliver bioactive proteins over long time periods.

The most relevant strategies are referenced below. Drug release from PLGA nano/microparticles can be controlled by the polymer molecular weight and the relation between monomers (lactide/glycolide) so that an increase in glycolic acid accelerates the weight loss of polymer due to the higher hydrophilicity of the matrix [75]. A mixture of different PLGA nanoparticles obtained using 50 : 50 and 75 : 50 latide/glycolide ratio has shown a great potential for protein drug delivery with a higher initial burst from PLGA 50 : 50. A slow release period has been observed for PLGA 75 : 50 encapsulating a glycoprotein (*α*-1-antitrypsin) with clinic activity in some pulmonary diseases [60].

On the other hand, a faster erosion of the microspheres with reduction in the PLGA molecular weight due to the facility of water penetration and the subsequent polymer degradation has been described [83]. Schrier et al. working with microspheres prepared by w/o/w using different types of PLGA analyzed the important role of the molecular weight, lactide-glycolide relation, and acid residues [57]. The amount of rhBMP2 adsorbed on the microparticle surface increased with the hydrophobicity of the polymer. At the same time, the release was in correlation with the degradation profile of the different polymers [57].

Thus, the use of more hydrophilic polymers reduces the hydrophobic protein-polymer interaction. This effect favors a more homogeneous distribution in the polymer matrix and increases the water uptake in the microspheres. Thus, the release rate of rhBMP2 encapsulated in microspheres composed by a PEG-PLGA di-block copolymer is increased with the PEG content of the polymer matrix [58]. A similar result was obtained using PLGA-PEG-PLGA triblock copolymers [59]. In this case, modifying the monomer relation (lactide-glycolide) in the PLGA and increasing the amount of PLGA-PEG-PLGA in the formulations, the release profile of BMP-2 coencapsulated with human SA in microespheres was adjustable. Similarly, the interaction of lysozyme with poloxamer 188 before their encapsulation produces a sustained release over 3 weeks without any burst effect. In the same line, using PLGA-PEG-PLGA as polymer, a sustained release of bioactive lysozime was extended over 45 days when the protein was complexed with poloxamer 188 previously to the encapsulation [120]. However, the presence of PEG300 as an additive of the inner phase of microparticles during the encapsulation process also influences the protein distribution and the release profile. In this case there is a decrease of the initial burst but with less overall release [58].

On the other hand, the use of PLGA-poloxamers blends is useful to obtain a sustained release for more than one month without any incidence in the high initial burst [56, 92]. However, for an encapsulated plasmid inside nanoparticles obtained by PLGA-poloxamer blends, the hydrophobicity of the surfactant allows prolonging the release up to 2 weeks in a controlled manner. Moreover, a complete release was reached for the PLGA-poloxamer blend instead PLGA nanoparticles, in which the maximum release was around 40% [84].

PLGA and poloxamers (pluronic F68) blends can also be used to obtain nanocomposite vesicles by a double emulsion process. These vesicles are suitable for the encapsulation of hydrophobic and hydrophilic molecules. The presence of pluronic affects the colloidal stability of the vesicles and the release pattern of the encapsulated molecules. These vesicles present a wall of 30 nm and the drug is encapsulated in the presence of the poloxamer [121].

Other strategies include the use of different compounds to increase the release time. Thus, BMP2 encapsulated in PLGA-PVA nanoparticles (around 300 nm) showed higher encapsulation efficiency and a short-time release profile with a very high initial burst. However, with the same synthesis procedure (w/o/w) but using PHBV (Poly(3 hydroxybutyrateco-3-hydroxivalerate)), BMP7 loaded nanocapsules had less encapsulation efficiency despite a long-time delivery. Nevertheless, the maximum released amount was lower. This difference in the release profile was due to the difference in hydrophilicity and degradation rates of both polymers [122]. Similarly, PLGA-poloxamer blend nanoparticles were superficially modified by introducing chitosan in the second step of the synthesis. This method showed a sustained release profile for up to 14 days without any initial important burst. In this case, a recombinant hepatitis B antigen was used [106]. Moreover, the use of heparin conjugated with PLGA porous microspheres has also been described to obtain a long-time delivery system reducing at the same time the initial burst. In these systems, heparin was immobilized onto the nano/microparticle surface. The release was controlled by using the binding affinities of heparin to several growth factors including BMP2. In this case, the initial burst was reduced to 4–7% during first day followed by a sustained release of about 1% per day [51–53].

The initial burst release may be attenuated by the fabrication of double-wall microspheres, that is, core-shell microparticles. The presence of a PLA shell reduces the release rate of BSA encapsulated in the PLGA core and extends the duration of the release profile up to two months. Moreover, an increase in the PLA molecular weight influences the rate of particle erosion, which further slows the protein release [123].

The modification of the viscosity in the environment of microparticles additionally influences the release pattern. Viscosity can control the burst at earliest time point and promote a sustained release. This situation has been shown for rhBMP2-PLGA microspheres embedded in a chitosan-thioglycolic acid hydrogel (Poloxamer 407) [124]. Yilgor et al. also incorporated the nanoparticles of their sequential delivery system into a scaffold composed by chitosan and chitosan-PEO [54]. In other work, PLGA/PVA microspheres with encapsulated BMP2 were combined with different composite biomaterials (gelatin hydrogel or polypropylene fumarate). The sustained release of the bioactive molecule was extended over a period of 42 days.* In vivo* results indicate the importance of the composite characteristics. In this case, an enhanced bone formation was obtained when the PLGA microparticles were incorporated into the more hydrophobic matrix (polypropylene fumarate) [125, 126].

Finally, Table 2 summarizes important information about different parameters related to the use of PLGA based nano- or microparticles to encapsulate, transport, and release growth factors (mainly BMP2).

### 3.6. Gene Therapy for Bone Tissue Engineering: Directed Delivery

In the last years, gene therapy has begun to play a role in bone tissue regeneration becoming an alternative method for the delivery of BMP2 [127, 128]. Thus, the genes encoding a specific protein can be delivered to a specific cell, rather than the proteins themselves. To reach this purpose, an efficient gene vector is necessary. Viral vectors possess the best transfection efficiency but numerous disadvantages, the most notable of them being the risk of mutagenesis. Nonviral vectors elude these problems but with a significant reduction in the transfection rate [129]. Therefore, intracellular delivery of bioactive agents has become the most used strategy for gene therapy, looking for the adequate transfection and consequent expression of the desired protein [79].

PLGA microspheres obtained by a w/o/w double emulsion process have been used by Qiao et al. to entrap plasmid-BMP2/polyethyleneimine nanoparticles. In this case, a sustained release of these nanoparticles until 35 days without initial burst was found resulting in differentiation of osteoblast promoted by the correct transfection of the delivered biofunctional BMP2-DNA [130].

In spite of the general caution with gene therapy, the genetic delivery of BMP2 has the potentiality of a better safety compared with the delivery of large amounts of recombinant protein [131]. Lu et al. specify the urgent need to develop more efficient delivery nanoparticles and transfection methods in order to apply the nonviral vectors in stem cell engineering and bone regeneration. Although enhanced bone formation has been shown in several recent studies using genes such as HIF-1*α* and miRNAs, new genetic sequences will be discovered and used in bone engineering in the near future that will most likely change our perspective [132].

PLGA nanospheres represent a well-studied biomolecule delivery system that could be applied to cell targeting, in order to enhance the delivery of specific proteins or nucleic acids inside or near the bone engineering reference cells, that is, mesenchymal stem cells [133]. The targeting properties can be supplied by a ligand functionalization strategy: modification of the surface structure of the nanocarrier by conjugating a cell-specific ligand to direct the release of encapsulated biomolecules preferably in close association with the target cells [134]. The use of pegylated nanoparticles with a covalent attachment of different ligands is reported as a potential technique to deliver bone cell-specific biomolecules for bone engineering [135].

Specific antibodies that recognize surface receptors in these cells could be covalently coupled to the surface of PLGA nanoparticles, obtaining “immunonanoparticles.” There are several examples of antibody immobilization on surface of PLGA nanoparticles. Kocbek et al. demonstrated the specific recognition of breast tumor cells by a specific monoclonal antibody attached on PLGA fluorescent nanoparticles obtained by W/O/W emulsion process [136]. For the surface covalent attachment, they used a more simple carbodiimide method, which promotes the formation of an amide bond between free carboxylic end groups of PLGA nanoparticles and primary amine groups of the antibody molecule [81]. This procedure can be highly influenced by the presence of stabilizers frequently used to confer colloidal stability to nanoparticles. The electrophoretic mobility of PLGA nanoparticles with an antibody (immuno-*γ*-globuline antihuman C-reactive protein) covalently attached on the surface is shown in Figure 5. It is necessary to remark the drastic decrease in the mobility values of the antibody-modified nanoparticles with respect to bare PLGA nanoparticles, which could imply low colloidal stability and the subsequent aggregation of the nanosystem. Santander-Ortega et al. proposed a lower antibody loading in which the bare PLGA patches must be coated by a nonionic surfactant in order to obtain immunoreactive stable nanoparticles [95]. Ratzinger et al. indicated that the presence of high poloxamer concentrations decreased the coupling efficiency to carboxylic end groups in PLGA nanoparticles, showing that an equilibrium that combines sufficient stability and the best coupling efficiency is necessary [98]. To prevent this problem, Cheng et al. synthetized carboxyl functionalized PLGA-PEG block copolymer, attaching a specific aptamer to the surface of pegylated nanoparticles via carbodiimide method. In this work, an enhanced drug delivery to prostate tumors has been shown in comparison to equivalent nontargeted nanoparticles [137].

### 3.7. Scaffolds

The data reported in the literature indicate that PLGA micro/nanoparticles are promising to achieve a sustained, spatial, and temporally controlled delivery of growth factors required for cell growth and cell differentiation. They can be incorporated with cells in solid scaffold or injectable hydrogels [73]. Scaffolds are porous 3D structures normally used to improve tissue-engineered bone [28]. According to Tian et al. [45], a scaffold designed with this objective must have (1) appropriate mechanical strength to support the growth of new bone; (2) appropriate porosity to allow ingrowth of bone-related cells; (3) good biocompatibility allowing the growth of cells on its surface without being rejected by the body; and (4) low toxicity to cells and tissues surrounded and (5) must be able to induce osteogenic differentiation of bone-related stem cells and (6) be biodegradable with nontoxic degradation products that can be eventually replaced by new bone. Additionally, the scaffold for bone regeneration must maintain the delivery or release of BMP (growth factors) “*in situ*” for a long time. In this way, nano/microparticles inside scaffolds are being used to release an adequate flow of these signaling biomolecules and preserve their functional structure [138]. The incorporation of colloidal micro/nanoparticles into fibrous scaffolds adds in the possibility of multiple drugs loading. However, this multidrug system could also involve a decrease of the mechanical properties of the structure and a possible loss of nanoparticles entrapped between the fibers [139]. Considering that the* in vivo* half-life of most biomolecules, especially proteins, is relatively short, it is essential that bioactive scaffolds maintain a desired concentration “*in situ*” to direct tissue regeneration. To do so, an initial release of the encapsulated growth factor in the first hours to quickly get an effective therapeutic concentration followed by a sustained long-time release profile is required [139]. Most of the polymeric particles inserted in scaffold structures are in a micron-scale. The main objective of these microparticles is the protection and temporary control of growth factor delivery. However, given the porosity of these structures, nanoparticles and especially particles of a few microns may become more important since it is possible to design systems with a simple and easy diffusion through the structure. This process could allow the specific recognition of a particular cell type, releasing their encapsulated BMPs in the same environment and helping their differentiation to cell/bone tissue. In any case, the larger-size microspheres might not necessarily be useless for bone regeneration scaffolds. As the microspheres gradually degrade, the space they occupied will be conducive to ingrowth of tissue. In addition to affecting the compression modulus of scaffolds because of their hollow feature, the particle size of microspheres can also influence the release of rhBMP2 [45].

## 4. Conclusion

The use of polymeric particles using PLGA is a promising system for a spatially and temporally controlled delivery of growth factors that promote cell growth and differentiation in bone engineering and regeneration by means of their incorporation beside cells into solid scaffold or hydrogels.

The PLGA is widely used for its biodegradability and biocompatibility and is approved by FDA and the European Medicines Agency for use in drug delivery systems supplied via parenteral. On the other hand, BMPs are potent growth factors for bone repair and specifically BMP2 shows excellent ability to induce bone formation of adequate quality. The procedure for synthesizing PLGA nano- or microparticles can be modified in their different variables to obtain systems with controlled size, in which it is possible to encapsulate hydrophobic or hydrophilic molecules, with an adequate colloidal stability and the possibility of surface functionalization for targeted delivery.

With this scenario, an optimization of methods and components must balance the structure and morphology of PLGA micro/nanoparticles in order to achieve high encapsulation efficiency of BMP2 and looking for a main goal: control of delivery, reducing the initial burst, and reaching a sustained release profile, preserving the biological activity, and directed to the target cells to minimize the clinical amount needed and allowing a correct bone tissue regeneration.

## Figures and Tables

**Table 1:** Summary of clinical and animal studies using BMP-2 for sinus floor elevation (adapted from [38]). The included studies overall concluded that rhBMP-2 induces new bone formation with comparable bone quality and quantity of newly formed bone to that induced by autogenous bone graft.

Reference	Study design	Follow-up (months)	Species (subjects)	Core biopsy harvesting (months)	Graft material	% Newly formed bone	Bone height gain (mm)	Bone width gain (mm)	Bone density (mg/mL)	Immune response	Histology
Boyne et al. 2005 [37]	RCT	52	Human (48)	6–11	0.75 mg/mL rhBMP-2/ACS	NA	11.29	Crest: 2.02 Midpoint: 8.54 Apical: 11.86	84	None	NA
					1.50 mg/mL rhBMP-2/ACS		0.47	Crest: 1.98 Midpoint: 7.80 Apical: 10.78	134		
					Autogenous bone graft; autogenous bone graft + allogeneic bone graft		10.16	Crest: 4.66 Midpoint: 10.17 Apical: 10.56	350		

Triplett et al. 2009 [40]	RCT	58	Human (160)	6	1.50 mg/mL rhBMP-2/ACS	NA	7.83 ± 3.52	NA	200	None	Rich vascular marrow space high in cellular content
					Autogenous bone graft (iliac crest, tibia or oral cavity); autogenous bone graft + allogeneic bone graft		9.46 ± 4.11		283		Osteoclasts still present; higher fibrous tissue

Kao et al. 2012 [41]	Prospective	6–9	Human (22)	6–9	rhBMP-2/ACS + ABB	16.04 ± 7.45	NA	NA	NA	None	Fewer ABB particles; less newly formed bone (woven and mature bone structure)
					ABB	24.85 ± 5.82					More ABB particles remaining; higher newly formed bone (woven and matured bone structure)

Nevins et al. 1996 [36]	Prospective	12	Goat (6)	12	rhBMP-2/ACS	NA	NA	NA	NA	None	Dense isolated trabeculae and bone marrow; osteoblast and osteoclasts; no cortical bone
					ACS/Buffer						Collagenous connective tissue; no evidence of inflammation; no neo-osteogenesis

Hanisch et al. 1997 [42]	RCT	24	Nonhuman primate (12)	24	rhBMP-2/ACS	NA	6.0 ± 0.3	NA	14.4 ± 2.9	NA	Newly formed bone indistinguishable from residual bone
					ACS		2.6 ± 0.3		13.9 ± 4.6		

Wada et al. 2001 [43]	Prospective	8	Rabbit (10)	8	rhBMP-2/ACS	22.4 ± 4.4	NA	NA	NA	NA	Cortical bone formation in both groups; trabeculae with clear lamellar structure were embedded in fatty marrow
					Autogenous bone graft (iliac crest)	21.9 ± 4.5					

Lee et al. 2013 [39]	Prospective	8	Mini-pig (8)	8	rhBMP-2/ACS	NA	9.3 ± 0.5	NA	51.9 ± 3	NA	Newly formed cancellous bone; new bone continuous with resident bone; woven bone in fibrovascular and fatty marrow
					Autogenous bone graft (iliac crest)		8.6 ± 0.7		32.9 ± 2.5		Irregular and variable bone among different subjects

NA: not available; RCT: randomized clinical trial; ACS: absorbable collagen sponge; ABB: anorganic bovine bone.

**Table 2:** Nano/microparticles systems to encapsulate GFs, mainly BMP2 growth factor. Most of them are in the microscopic scale and were used to be entrapped into scaffold of different characteristics. PVA has been the more used surfactant-stabilizer. It is possible to find both, encapsulation and surface adsorption of the growth factors with high-moderate efficiency. The use of heparin as stabilizer reduces significantly the initial burst release, favoring a sustained release in the time. The bioactivity of the GF was preserved in most of the systems and coencapsulation with other biomolecules seems to have a similar effect than the use of surfactants as stabilizers.

Polymers	Stabilizer	Size	Encapsulation % EE	Release	Biological activity	Reference
PLGA	PVA	10–20 *µ*m	Adsorbed rhBMP2	20 ng/mL of constant sustained release	Better bone formation after 8 weeks	Fu et al. 2013 [44]

PLGA	PVA	10–100 *μ*m	rhBMP2-BSA 69% (BMP)	Burst (20%) Sustained until 77% (28 days)	BMP2 molecules with bioactivity	Tian et al. 2012 [45]

PLGA 75 : 25	PVA	182 *μ*m	82%	—	Good bone defect repair outcomes within 8−12 weeks	Rodríguez-Évora et al. 2014 [46]

PLGA	PVA	228 *μ*m	60,5%	30% initial burst. Slower release of 4% per week. After 8 weeks 60% released	No loss of bioactivity	Reyes et al. 2013 [47]

PLGA/PEG	No double emulsion synthesis	100–200 *μ*m	Adsorbed BMP2	13% initial burst. Slower release of 0.01–8% per day. After 23 days 70% released	Substantial bone regeneration of the scaffold	Rahman et al. 2014 [48]

Different PLGA	PVA	20–100 *μ*m	30% (uncapped PLGA) 90% (capped PLGA)	26–49% (1 day) Total after 2 weeks	No loss of bioactivity	Lupu-Haber et al. 2013 [49]

PLGA 75 : 25	PVA	5–125 *μ*m	—	Initial burst 30% (1 day) Sustained 35 days	Higher volumes and surface area coverage of new bone	Wink et al. 2014 [50]

PLGA	Heparin	200–800 nm	Adsorbed BMP2 94%	No initial burst. Sustained over 4 weeks	Significant reduction of the BMP2 dose for good bone formation	La et al. 2010 [51]

PLGA	Heparin- Poloxamer	160 nm	Adsorbed BMP2 100%	Initial burst (4–7%) linear profile	Higher matrix mineralization of regenerated bone	Chung et al. 2007 [52]

PLGA	Heparin	100–250 nm	Adsorbed 94%	Initial burst 10% (1 day) 60% after 30 days	No loss of bioactivity Efficacy of administration, amount 50-fold lower	Jeon et al. 2008 [53]

PLGA	PVA	~300 nm	80%	85% initial burst (1 day)	No loss of bioactivity	Yilgor et al. 2009 [54]

PLGA (in rings)	PVA	215 *μ*m	66%	Moderate burst Sustained release over 6 weeks	60% of calvaria defect were healed	Rodríguez-Évora et al. 2013 [55]

PLGA-Poloxamer 188 Blend	Poloxamer	150 nm	FGF-BSA-Heparin 60–80%	40% initial burst (1 day), 60% (30 days)	No loss of bioactivity	d'Angelo et al. 2010 [56]

Different PLGA polymers	PVA	*μ*m order	rhBMP2 adsorption 40–75%	20–80% initial burst (1 day)	—	Schrier et al. 2001 [57]

PLGA/PEG	PVA	37–67 *μ*m	72–99%	33% initial burst (1 day)	Little loss of bioactivity	Lochmann et al. 2010 [58]

PLGA/PLGA-PEG-PLGA	PVA	100 *μ*m	HSA-BMP2 60%	70% initial burst (1 day)	No loss of bioactivity	White et al. 2013 [59]

PLGA	PVA	100–1000 nm	*Α*-1-antitrypsin 90%	30% initial burst (1 day) 50% after 24 days	Biological activity was preserved using BSA and *β*-cyclodextrine.	Pirooznia et al. 2012 [60]

## References

[1] Padial-Molina M., Galindo-Moreno P., Avila-Ortiz G. (2009). Biomimetic ceramics in implant dentistry. *Minerva Biotecnologica*.

[2] Al-Nawas B., Schiegnitz E. (2014). Augmentation procedures using bone substitute materials or autogenous bone—a systematic review and meta-analysis. *European Journal of Oral Implantology*.

[3] Katranji A., Fotek P., Wang H.-L. (2008). Sinus augmentation complications: etiology and treatment. *Implant Dentistry*.

[4] Misch C. E. (1987). Maxillary sinus augmentation for endosteal implants: organized alternative treatment plans. *The International Journal of Oral Implantology: Implantologist*.

[5] Myeroff C., Archdeacon M. (2011). Autogenous bone graft: donor sites and techniques. *The Journal of Bone & Joint Surgery—American Volume*.

[6] Avila G., Neiva R., Misch C. E. (2010). Clinical and histologic outcomes after the use of a novel allograft for maxillary sinus augmentation: a case series. *Implant Dentistry*.

[7] Froum S. J., Wallace S. S., Elian N., Cho S. C., Tarnow D. P. (2006). Comparison of mineralized cancellous bone allograft (Puros) and anorganic bovine bone matrix (Bio-Oss) for sinus augmentation: histomorphometry at 26 to 32 weeks after grafting. *The International Journal of Periodontics & Restorative Dentistry*.

[8] Galindo-Moreno P., Ávila G., Fernández-Barbero J. E. (2007). Evaluation of sinus floor elevation using a composite bone graft mixture. *Clinical Oral Implants Research*.

[9] Galindo-Moreno P., Moreno-Riestra I., Avila G. (2011). Effect of anorganic bovine bone to autogenous cortical bone ratio upon bone remodeling patterns following maxillary sinus augmentation. *Clinical Oral Implants Research*.

[10] Wheeler S. L. (1997). Sinus augmentation for dental implants: the use of alloplastic materials. *Journal of Oral and Maxillofacial Surgery*.

[11] Wallace S. S., Froum S. J. (2003). Effect of maxillary sinus augmentation on the survival of endosseous dental implants. A systematic review.. *Annals of Periodontology/the American Academy of Periodontology*.

[12] Padial-Molina M., Rios H. F. (2014). Stem cells, scaffolds and gene therapy for periodontal engineering. *Current Oral Health Reports*.

[13] Padial-Molina M., Volk S. L., Rios H. F. (2014). Periostin increases migration and proliferation of human periodontal ligament fibroblasts challenged by tumor necrosis factor -alpha and *Porphyromonas gingivalis* lipopolysaccharides. *Journal of Periodontal Research*.

[14] Behnia H., Khojasteh A., Soleimani M., Tehranchi A., Atashi A. (2012). Repair of alveolar cleft defect with mesenchymal stem cells and platelet derived growth factors: a preliminary report. *Journal of Cranio-Maxillofacial Surgery*.

[15] Padial-Molina M., Marchesan J. T., Taut A. D., Jin Q., Giannobile W. V., Rios H. F. (2012). Methods to validate tooth-supporting regenerative therapies. *Methods in Molecular Biology*.

[16] Urist M. R. (1965). Bone: formation by autoinduction. *Science*.

[17] Boyne P., Jones S. D. (2004). Demonstration of the osseoinductive effect of bone morphogenetic protein within endosseous dental implants. *Implant Dentistry*.

[18] Wang E. A., Rosen V., D'Alessandro J. S. (1990). Recombinant human bone morphogenetic protein induces bone formation. *Proceedings of the National Academy of Sciences of the United States of America*.

[19] Wozney J. M. (1992). The bone morphogenetic protein family and osteogenesis. *Molecular Reproduction and Development*.

[20] Barboza E., Caúla A., Machado F. (1999). Potential of recombinant human bone morphogenetic protein-2 in bone regeneration. *Implant Dentistry*.

[21] Carreira A. C., Alves G. G., Zambuzzi W. F., Sogayar M. C., Granjeiro J. M. (2014). Bone morphogenetic proteins: structure, biological function and therapeutic applications. *Archives of Biochemistry and Biophysics*.

[22] Bustos-Valenzuela J. C., Fujita A., Halcsik E., Granjeiro J. M., Sogayar M. C. (2011). Unveiling novel genes upregulated by both rhBMP2 and rhBMP7 during early osteoblastic transdifferentiation of C2C12 cells. *BMC Research Notes*.

[23] Tsuji K., Bandyopadhyay A., Harfe B. D. (2006). BMP2 activity, although dispensable for bone formation, is required for the initiation of fracture healing. *Nature Genetics*.

[24] Tsuji K., Cox K., Bandyopadhyay A., Harfe B. D., Tabin C. J., Rosen V. (2008). BMP4 is dispensable for skeletogenesis and fracture-healing in the limb. *The Journal of Bone and Joint Surgery—American Volume*.

[25] Tsuji K., Cox K., Gamer L., Graf D., Economides A., Rosen V. (2010). Conditional deletion of BMP7 from the limb skeleton does not affect bone formation or fracture repair. *Journal of Orthopaedic Research*.

[26] Chen G., Deng C., Li Y.-P. (2012). TGF-beta and BMP signaling in osteoblast differentiation and bone formation. *International Journal of Biological Sciences*.

[27] Ramel M.-C., Hill C. S. (2012). Spatial regulation of BMP activity. *FEBS Letters*.

[28] Carreira A. C., Lojudice F. H., Halcsik E., Navarro R. D., Sogayar M. C., Granjeiro J. M. (2014). Bone morphogenetic proteins: facts, challenges, and future perspectives. *Journal of Dental Research*.

[29] Deschaseaux F., Sensébé L., Heymann D. (2009). Mechanisms of bone repair and regeneration. *Trends in Molecular Medicine*.

[30] Mueller T. D., Nickel J. (2012). Promiscuity and specificity in BMP receptor activation. *FEBS Letters*.

[31] Sieber C., Kopf J., Hiepen C., Knaus P. (2009). Recent advances in BMP receptor signaling. *Cytokine & Growth Factor Reviews*.

[32] Sapkota G., Alarcón C., Spagnoli F. M., Brivanlou A. H., Massagué J. (2007). Balancing BMP signaling through integrated inputs into the Smad1 linker. *Molecular Cell*.

[33] McKay W. F., Peckham S. M., Badura J. M. (2007). A comprehensive clinical review of recombinant human bone morphogenetic protein-2 (INFUSE Bone Graft). *International Orthopaedics*.

[34] Hong P., Boyd D., Beyea S. D., Bezuhly M. (2013). Enhancement of bone consolidation in mandibular distraction osteogenesis: a contemporary review of experimental studies involving adjuvant therapies. *Journal of Plastic, Reconstructive and Aesthetic Surgery*.

[35] Spagnoli D. B., Marx R. E. (2011). Dental implants and the use of rhBMP-2. *Dental Clinics of North America*.

[36] Nevins M., Kirker-Head C., Nevins M., Wozney J. A., Palmer R., Graham D. (1996). Bone formation in the goat maxillary sinus induced by absorbable collagen sponge implants impregnated with recombinant human bone morphogenetic protein-2. *The International Journal of Periodontics & Restorative Dentistry*.

[37] Boyne P. J., Lilly L. C., Marx R. E. (2005). De novo bone induction by recombinant human bone morphogenetic protein-2 (rhBMP-2) in maxillary sinus floor augmentation. *Journal of Oral and Maxillofacial Surgery*.

[38] Torrecillas-Martinez L., Monje A., Pikos M. A. (2013). Effect of rhBMP-2 upon maxillary sinus augmentation: a comprehensive review. *Implant Dentistry*.

[39] Lee J., Susin C., Rodriguez N. A. (2013). Sinus augmentation using rhBMP-2/ACS in a mini-pig model: relative efficacy of autogenous fresh particulate iliac bone grafts. *Clinical Oral Implants Research*.

[40] Triplett R. G., Nevins M., Marx R. E. (2009). Pivotal, randomized, parallel evaluation of recombinant human bone morphogenetic protein-2/absorbable collagen sponge and autogenous bone graft for maxillary sinus floor augmentation. *Journal of Oral and Maxillofacial Surgery*.

[41] Kao D. W. K., Kubota A., Nevins M., Fiorellini J. P. (2012). The negative effect of combining rhBMP-2 and Bio-Oss on bone formation for maxillary sinus augmentation. *The International Journal of Periodontics & Restorative Dentistry*.

[42] Hanisch O., Tatakis D. N., Rohrer M. D., Wöhrle P. S., Wozney J. M., Wikesjö U. M. E. (1997). Bone formation and osseointegration stimulated by rhBMP-2 following subantral augmentation procedures in nonhuman primates. *The International Journal of Oral & Maxillofacial Implants*.

[43] Wada K., Niimi A., Watanabe K., Sawai T., Ueda M. (2001). Maxillary sinus floor augmentation in rabbits: a comparative histologic-histomorphometric study between rhBMP-2 and autogenous bone. *The International Journal of Periodontics & Restorative Dentistry*.

[44] Fu R., Selph S., McDonagh M. (2013). Effectiveness and harms of recombinant human bone morphogenetic protein-2 in spine fusion: a systematic review and meta-analysis. *Annals of Internal Medicine*.

[45] Tian Z., Zhu Y., Qiu J. (2012). Synthesis and characterization of UPPE-PLGA-rhBMP2 scaffolds for bone regeneration. *Journal of Huazhong University of Science and Technology—Medical Science*.

[46] Rodríguez-Évora M., García-Pizarro E., del Rosario C. (2014). Smurf1 knocked-down, mesenchymal stem cells and BMP-2 in an electrospun system for bone regeneration. *Biomacromolecules*.

[47] Reyes R., Delgado A., Solis R. (2013). Cartilage repair by local delivery of TGF-*β*1 or BMP-2 from a novel, segmented polyurethane/polylactic-co-glycolic bilayered scaffold. *Journal of Biomedical Materials Research Part A*.

[48] Rahman C. V., Ben-David D., Dhillon A. (2014). Controlled release of BMP-2 from a sintered polymer scaffold enhances bone repair in a mouse calvarial defect model. *Journal of Tissue Engineering and Regenerative Medicine*.

[49] Lupu-Haber Y., Pinkas O., Boehm S., Scheper T., Kasper C., Machluf M. (2013). Functionalized PLGA-doped zirconium oxide ceramics for bone tissue regeneration. *Biomedical Microdevices*.

[50] Wink J. D., Gerety P. A., Sherif R. D. (2014). Sustained delivery of rhBMP-2 by means of poly(lactic-co-glycolic acid) microspheres: cranial bone regeneration without heterotopic ossification or craniosynostosis. *Plastic and Reconstructive Surgery*.

[51] La W.-G., Kang S.-W., Yang H. S. (2010). The efficacy of bone morphogenetic protein-2 depends on its mode of delivery. *Artificial Organs*.

[52] Chung Y.-I., Ahn K.-M., Jeon S.-H., Lee S.-Y., Lee J.-H., Tae G. (2007). Enhanced bone regeneration with BMP-2 loaded functional nanoparticle-hydrogel complex. *Journal of Controlled Release*.

[53] Jeon O., Song S. J., Yang H. S. (2008). Long-term delivery enhances in vivo osteogenic efficacy of bone morphogenetic protein-2 compared to short-term delivery. *Biochemical and Biophysical Research Communications*.

[54] Yilgor P., Tuzlakoglu K., Reis R. L., Hasirci N., Hasirci V. (2009). Incorporation of a sequential BMP-2/BMP-7 delivery system into chitosan-based scaffolds for bone tissue engineering. *Biomaterials*.

[55] Rodríguez-Évora M., Delgado A., Reyes R. (2013). Osteogenic effect of local, long versus short term BMP-2 delivery from a novel SPU-PLGA-*β*TCP concentric system in a critical size defect in rats. *European Journal of Pharmaceutical Sciences*.

[56] d'Angelo I., Garcia-Fuentes M., Parajó Y. (2010). Nanoparticles based on PLGA: poloxamer blends for the delivery of proangiogenic growth factors. *Molecular Pharmaceutics*.

[57] Schrier J. A., Fink B. F., Rodgers J. B., Vasconez H. C., DeLuca P. P. (2001). Effect of a freeze-dried CMC/PLGA microsphere matrix of rhBMP-2 on bone healing. *AAPS PharmSciTech*.

[58] Lochmann A., Nitzsche H., von Einem S., Schwarz E., Mäder K. (2010). The influence of covalently linked and free polyethylene glycol on the structural and release properties of rhBMP-2 loaded microspheres. *Journal of Controlled Release*.

[59] White L. J., Kirby G. T. S., Cox H. C. (2013). Accelerating protein release from microparticles for regenerative medicine applications. *Materials Science and Engineering C: Materials for Biological Applications*.

[60] Pirooznia N., Hasannia S., Lotfi A. S., Ghanei M. (2012). Encapsulation of alpha-1 antitrypsin in PLGA nanoparticles: in vitro characterization as an effective aerosol formulation in pulmonary diseases. *Journal of Nanobiotechnology*.

[61] Ronga M., Fagetti A., Canton G., Paiusco E., Surace M. F., Cherubino P. (2013). Clinical applications of growth factors in bone injuries: experience with BMPs. *Injury*.

[62] Devine J. G., Dettori J. R., France J. C., Brodt E., McGuire R. A. (2012). The use of rhBMP in spine surgery: is there a cancer risk?. *Evidence-Based Spine-Care Journal*.

[63] Carragee E. J., Hurwitz E. L., Weiner B. K. (2011). A critical review of recombinant human bone morphogenetic protein-2 trials in spinal surgery: emerging safety concerns and lessons learned. *The Spine Journal*.

[64] Simmonds M. C., Brown J. V. E., Heirs M. K. (2013). Safety and effectiveness of recombinant human bone morphogenetic protein-2 for spinal fusion: a meta-analysis of individual-participant data. *Annals of Internal Medicine*.

[65] Chung V. H.-Y., Chen A. Y.-L., Jeng L.-B., Kwan C.-C., Cheng S.-H., Chang S. C.-N. (2012). Engineered autologous bone marrow mesenchymal stem cells: alternative to cleft alveolar bone graft surgery. *Journal of Craniofacial Surgery*.

[66] Ratko T. A., Belinson S. E., Samson D. J., Bonnell C., Ziegler K. M., Aronson N. (2010). *Bone Morphogenetic Protein: The State of the Evidence of On-Label and Off-Label Use*.

[67] Barratt G. (2003). Colloidal drug carriers: achievements and perspectives. *Cellular and Molecular Life Sciences*.

[68] Bramwell V. W., Perrie Y. (2005). Particulate delivery systems for vaccines. *Critical Reviews in Therapeutic Drug Carrier Systems*.

[69] Csaba N., Garcia-Fuentes M., Alonso M. J. (2006). The performance of nanocarriers for transmucosal drug delivery. *Expert Opinion on Drug Delivery*.

[70] Santander-Ortega M. J., Stauner T., Loretz B. (2010). Nanoparticles made from novel starch derivatives for transdermal drug delivery. *Journal of Controlled Release*.

[71] Jiang W., Gupta R. K., Deshpande M. C., Schwendeman S. P. (2005). Biodegradable poly(lactic-co-glycolic acid) microparticles for injectable delivery of vaccine antigens. *Advanced Drug Delivery Reviews*.

[72] Shantha Kumar T. R., Soppimath K., Nachaegari S. K. (2006). Novel delivery technologies for protein and peptide therapeutics. *Current Pharmaceutical Biotechnology*.

[73] Danhier F., Ansorena E., Silva J. M., Coco R., Le Breton A., Préat V. (2012). PLGA-based nanoparticles: an overview of biomedical applications. *Journal of Controlled Release*.

[74] Kumari A., Yadav S. K., Yadav S. C. (2010). Biodegradable polymeric nanoparticles based drug delivery systems. *Colloids and Surfaces B: Biointerfaces*.

[75] Makadia H. K., Siegel S. J. (2011). Poly Lactic-co-Glycolic Acid (PLGA) as biodegradable controlled drug delivery carrier. *Polymers*.

[76] Mohamed F., van der Walle C. F. (2008). Engineering biodegradable polyester particles with specific drug targeting and drug release properties. *Journal of Pharmaceutical Sciences*.

[77] Silva G. A., Coutinho O. P., Ducheyne P., Reis R. L. (2007). Materials in particulate form for tissue engineering. 2. Applications in bone. *Journal of Tissue Engineering and Regenerative Medicine*.

[78] Zhang S., Uludag H. (2009). Nanoparticulate systems for growth factor delivery. *Pharmaceutical Research*.

[79] Santo V. E., Gomes M. E., Mano J. F., Reis R. L. (2012). From nano-to macro-scale: nanotechnology approaches for spatially controlled delivery of bioactive factors for bone and cartilage engineering. *Nanomedicine*.

[80] Tran M.-K., Swed A., Boury F. (2012). Preparation of polymeric particles in CO_2_ medium using non-toxic solvents: formulation and comparisons with a phase separation method. *European Journal of Pharmaceutics and Biopharmaceutics*.

[81] Ertl B., Heigl F., Wirth M., Gabor F. (2000). Lectin-mediated bioadhesion: preparation, stability and Caco-2 binding of wheat germ agglutinin-functionalized poly-(D,L-lactic-co-glycolic acid)-microspheres. *Journal of Drug Targeting*.

[82] Hans M. L., Lowman A. M. (2002). Biodegradable nanoparticles for drug delivery and targeting. *Current Opinion in Solid State and Materials Science*.

[83] Blanco D., Alonso M. J. (1998). Protein encapsulation and release from poly(lactide-co-glycolide) microspheres: effect of the protein and polymer properties and of the co-encapsulation of surfactants. *European Journal of Pharmaceutics and Biopharmaceutics*.

[84] Csaba N., Caamaño P., Sánchez A., Domínguez F., Alonso M. J. (2005). PLGA: poloxamer and PLGA:poloxamine blend nanoparticles: new carriers for gene delivery. *Biomacromolecules*.

[85] Sturesson C., Carlfors J. (2000). Incorporation of protein in PLG-microspheres with retention of bioactivity. *Journal of Controlled Release*.

[86] Rosca I. D., Watari F., Uo M. (2004). Microparticle formation and its mechanism in single and double emulsion solvent evaporation. *Journal of Controlled Release*.

[87] Meng F. T., Ma G. H., Qiu W., Su Z. G. (2003). W/O/W double emulsion technique using ethyl acetate as organic solvent: effects of its diffusion rate on the characteristics of microparticles. *Journal of Controlled Release*.

[88] Ghaderi R., Carlfors J. (1997). Biological activity of lysozyme after entrapment in poly (d,l-lactide-co-glycolide)-microspheres. *Pharmaceutical Research*.

[89] Wang H., Leeuwenburgh S. C. G., Li Y., Jansen J. A. (2012). The use of micro- and nanospheres as functional components for bone tissue regeneration. *Tissue Engineering Part B: Reviews*.

[90] Xiong S., Zhao X., Heng B. C., Ng K. W., Loo J. S.-C. (2011). Cellular uptake of Poly-(D,L-lactide-co-glycolide) (PLGA) nanoparticles synthesized through solvent emulsion evaporation and nanoprecipitation method. *Biotechnology Journal*.

[91] Chou L. Y. T., Ming K., Chan W. C. W. (2011). Strategies for the intracellular delivery of nanoparticles. *Chemical Society Reviews*.

[92] Santander-Ortega M. J., Lozano-López M. V., Bastos-González D., Peula-García J. M., Ortega-Vinuesa J. L. (2010). Novel core-shell lipid-chitosan and lipid-poloxamer nanocapsules: stability by hydration forces. *Colloid and Polymer Science*.

[93] Yang Y.-Y., Chung T.-S., Ping Ng N. (2001). Morphology, drug distribution, and in vitro release profiles of biodegradable polymeric microspheres containing protein fabricated by double-emulsion solvent extraction/evaporation method. *Biomaterials*.

[94] Feczkó T., Tóth J., Gyenis J. (2008). Comparison of the preparation of PLGA-BSA nano- and microparticles by PVA, poloxamer and PVP. *Colloids and Surfaces A: Physicochemical and Engineering Aspects*.

[95] Santander-Ortega M. J., Bastos-González D., Ortega-Vinuesa J. L. (2007). Electrophoretic mobility and colloidal stability of PLGA particles coated with IgG. *Colloids and Surfaces B: Biointerfaces*.

[96] Yang Y.-Y., Chia H.-H., Chung T.-S. (2000). Effect of preparation temperature on the characteristics and release profiles of PLGA microspheres containing protein fabricated by double-emulsion solvent extraction/evaporation method. *Journal of Controlled Release*.

[97] Fang D.-L., Chen Y., Xu B. (2014). Development of lipid-shell and polymer core nanoparticles with water-soluble salidroside for anti-cancer therapy. *International Journal of Molecular Sciences*.

[98] Ratzinger G., Länger U., Neutsch L., Pittner F., Wirth M., Gabor F. (2010). Surface modification of PLGA particles: the interplay between stabilizer, ligand size, and hydrophobic interactions. *Langmuir*.

[99] Feng S.-S., Huang G. (2001). Effects of emulsifiers on the controlled release of paclitaxel (Taxol) from nanospheres of biodegradable polymers. *Journal of Controlled Release*.

[100] Chan J. M., Zhang L., Yuet K. P. (2009). PLGA-lecithin-PEG core-shell nanoparticles for controlled drug delivery. *Biomaterials*.

[101] Fraylich M., Wang W., Shakesheff K., Alexander C., Saunders B. (2008). Poly(D,L-lactide-co-glycolide) dispersions containing pluronics: from particle preparation to temperature-triggered aggregation. *Langmuir*.

[102] Santander-Ortega M. J., Peula-García J. M., Goycoolea F. M., Ortega-Vinuesa J. L. (2011). Chitosan nanocapsules: effect of chitosan molecular weight and acetylation degree on electrokinetic behaviour and colloidal stability. *Colloids and Surfaces B: Biointerfaces*.

[103] Tan J. S., Butterfield D. E., Voycheck C. L., Caldwell K. D., Li J. T. (1993). Surface modification of nanoparticles by PEO/PPO block copolymers to minimize interactions with blood components and prolong blood circulation in rats. *Biomaterials*.

[104] Bouissou C., Potter U., Altroff H., Mardon H., van der Walle C. (2004). Controlled release of the fibronectin central cell binding domain from polymeric microspheres. *Journal of Controlled Release*.

[105] Gref R., Minamitake Y., Peracchia M. T., Trubetskoy V., Torchilin V., Langer R. (1994). Biodegradable long-circulating polymeric nanospheres. *Science*.

[106] Paolicelli P., Prego C., Sanchez A., Alonso M. J. (2010). Surface-modified PLGA-based nanoparticles that can efficiently associate and deliver virus-like particles. *Nanomedicine*.

[107] Brigger I., Dubernet C., Couvreur P. (2002). Nanoparticles in cancer therapy and diagnosis. *Advanced Drug Delivery Reviews*.

[108] Giteau A., Venier-Julienne M. C., Aubert-Pouëssel A., Benoit J. P. (2008). How to achieve sustained and complete protein release from PLGA-based microparticles?. *International Journal of Pharmaceutics*.

[109] Balmayor E. R., Feichtinger G. A., Azevedo H. S., van Griensven M., Reis R. L. (2009). Starch-poly-*ε*-caprolactone microparticles reduce the needed amount of BMP-2. *Clinical Orthopaedics and Related Research*.

[110] Santander-Ortega M. J., Bastos-González D., Ortega-Vinuesa J. L., Alonso M. J. (2009). Insulin-loaded PLGA nanoparticles for oral administration: an in vitro physico-chemical characterization. *Journal of Biomedical Nanotechnology*.

[111] Meinel L., Illi O. E., Zapf J., Malfanti M., Peter Merkle H., Gander B. (2001). Stabilizing insulin-like growth factor-I in poly(D,L-lactide-co-glycolide) microspheres. *Journal of Controlled Release*.

[112] Srinivasan C., Katare Y. K., Muthukumaran T., Panda A. K. (2005). Effect of additives on encapsulation efficiency, stability and bioactivity of entrapped lysozyme from biodegradable polymer particles. *Journal of Microencapsulation*.

[113] Tobío M., Schwendeman S. P., Guo Y., McIver J., Langer R., Alonso M. J. (1999). Improved immunogenicity of a core-coated tetanus toxoid delivery system. *Vaccine*.

[114] Malik D. K., Baboota S., Ahuja A., Hasan S., Ali J. (2007). Recent advances in protein and peptide drug delivery systems. *Current Drug Delivery*.

[115] Cleland J. L., Sanders L. M., Hendron R. W. (1997). Protein delivery from biodegradable microspheres. *Protein Delivery: Physical Systems*.

[116] Oh S. H., Kim T. H., Lee J. H. (2011). Creating growth factor gradients in three dimensional porous matrix by centrifugation and surface immobilization. *Biomaterials*.

[117] Brown B. N., Valentin J. E., Stewart-Akers A. M., McCabe G. P., Badylak S. F. (2009). Macrophage phenotype and remodeling outcomes in response to biologic scaffolds with and without a cellular component. *Biomaterials*.

[118] Brown B. N., Freund J. M., Han L. (2011). Comparison of three methods for the derivation of a biologic scaffold composed of adipose tissue extracellular matrix. *Tissue Engineering—Part C: Methods*.

[119] Li B., Yoshii T., Hafeman A. E., Nyman J. S., Wenke J. C., Guelcher S. A. (2009). The effects of rhBMP-2 released from biodegradable polyurethane/microsphere composite scaffolds on new bone formation in rat femora. *Biomaterials*.

[120] Paillard-Giteau A., Tran V. T., Thomas O. (2010). Effect of various additives and polymers on lysozyme release from PLGA microspheres prepared by an s/o/w emulsion technique. *European Journal of Pharmaceutics and Biopharmaceutics*.

[121] Nair B. P., Sharma C. P. (2012). Poly(lactide-*co*-glycolide)-laponite-F68 nanocomposite vesicles through a single-step double-emulsion method for the controlled release of doxorubicin. *Langmuir*.

[122] Yilgor P., Hasirci N., Hasirci V. (2010). Sequential BMP-2/BMP-7 delivery from polyester nanocapsules. *Journal of Biomedical Materials Research—Part A*.

[123] Xia Y., Xu Q., Wang C.-H., Pack D. W. (2013). Protein encapsulation in and release from monodisperse double-wall polymer microspheres. *Journal of Pharmaceutical Sciences*.

[124] Fu Y., Du L., Wang Q. (2012). In vitro sustained release of recombinant human bone morphogenetic protein-2 microspheres embedded in thermosensitive hydrogels. *Die Pharmazie*.

[125] Kempen D. H. R., Lu L., Hefferan T. E. (2008). Retention of in vitro and in vivo BMP-2 bioactivities in sustained delivery vehicles for bone tissue engineering. *Biomaterials*.

[126] Kempen D. H. R., Lu L., Heijink A. (2009). Effect of local sequential VEGF and BMP-2 delivery on ectopic and orthotopic bone regeneration. *Biomaterials*.

[127] Nie H., Ho M.-L., Wang C.-K., Wang C.-H., Fu Y.-C. (2009). BMP-2 plasmid loaded PLGA/HAp composite scaffolds for treatment of bone defects in nude mice. *Biomaterials*.

[128] Wegman F., van der Helm Y., Öner F. C., Dhert W. J. A., Alblas J. (2013). Bone morphogenetic protein-2 plasmid DNA as a substitute for bone morphogenetic protein-2 protein in bone tissue engineering. *Tissue Engineering Part A*.

[129] Fischer J., Kolk A., Wolfart S. (2011). Future of local bone regeneration—protein versus gene therapy. *Journal of Cranio-Maxillofacial Surgery*.

[130] Qiao C., Zhang K., Jin H. (2013). Using poly(lactic-co-glycolic acid) microspheres to encapsulate plasmid of bone morphogenetic protein 2/polyethylenimine nanoparticles to promote bone formation in vitro and in vivo. *International Journal of Nanomedicine*.

[131] Evans C. H. (2012). Gene delivery to bone. *Advanced Drug Delivery Reviews*.

[132] Lu C.-H., Chang Y.-H., Lin S.-Y., Li K.-C., Hu Y.-C. (2013). Recent progresses in gene delivery-based bone tissue engineering. *Biotechnology Advances*.

[133] Vo T. N., Kasper F. K., Mikos A. G. (2012). Strategies for controlled delivery of growth factors and cells for bone regeneration. *Advanced Drug Delivery Reviews*.

[134] Ji W., Wang H., van den Beucken J. J. J. P. (2012). Local delivery of small and large biomolecules in craniomaxillofacial bone. *Advanced Drug Delivery Reviews*.

[135] Luginbuehl V., Meinel L., Merkle H. P., Gander B. (2004). Localized delivery of growth factors for bone repair. *European Journal of Pharmaceutics and Biopharmaceutics*.

[136] Kocbek P., Obermajer N., Cegnar M., Kos J., Kristl J. (2007). Targeting cancer cells using PLGA nanoparticles surface modified with monoclonal antibody. *Journal of Controlled Release*.

[137] Cheng J., Teply B. A., Sherifi I. (2007). Formulation of functionalized PLGA-PEG nanoparticles for *in vivo* targeted drug delivery. *Biomaterials*.

[138] Romagnoli C., D'Asta F., Brandi M. L. (2013). Drug delivery using composite scaffolds in the context of bone tissue engineering. *Clinical Cases in Mineral and Bone Metabolism*.

[139] Puppi D., Zhang X., Yang L., Chiellini F., Sun X., Chiellini E. (2014). Nano/microfibrous polymeric constructs loaded with bioactive agents and designed for tissue engineering applications: a review. *Journal of Biomedical Materials Research Part B: Applied Biomaterials*.

